# Voices of care: unveiling patient journeys in primary care for hypertension and diabetes management in Kerala, India

**DOI:** 10.3389/fpubh.2024.1375227

**Published:** 2024-05-22

**Authors:** Ranjana Ravindranath, P. Sankara Sarma, Sivasubramonian Sivasankaran, Kavumpurathu Raman Thankappan, Panniyammakal Jeemon

**Affiliations:** ^1^Achutha Menon Centre for Health Science Studies, Sree Chitra Tirunal Institute for Medical Sciences and Technology, Trivandrum, Kerala, India; ^2^Manipal Academy of Higher Education, Manipal, Karnataka, India; ^3^Amrita Institute of Medical Sciences, Kochi, Kerala, India

**Keywords:** access, primary health, health needs, community, NCD, qualitative research, health care pathway

## Abstract

**Background:**

Diabetes and hypertension are leading public health problems, particularly affecting low- and middle-income countries, with considerable variations in the care continuum between different age, socio-economic, and rural and urban groups. In this qualitative study, examining the factors affecting access to healthcare in Kerala, we aim to explore the healthcare-seeking pathways of people living with diabetes and hypertension.

**Methods:**

We conducted 20 semi-structured interviews and one focus group discussion (FGD) on a purposive sample of people living with diabetes and hypertension. Participants were recruited at four primary care facilities in Malappuram district of Kerala. Interviews were transcribed and analyzed deductively and inductively using thematic analysis underpinned by Levesque et al.’s framework.

**Results:**

The patient journey in managing diabetes and hypertension is complex, involving multiple entry and exit points within the healthcare system. Patients did not perceive Primary Health Centres (PHCs) as their initial points of access to healthcare, despite recognizing their value for specific services. Numerous social, cultural, economic, and health system determinants underpinned access to healthcare. These included limited patient knowledge of their condition, self-medication practices, lack of trust/support, high out-of-pocket expenditure, unavailability of medicines, physical distance to health facilities, and attitude of healthcare providers.

**Conclusion:**

The study underscores the need to improve access to timely diagnosis, treatment, and ongoing care for diabetes and hypertension at the lower level of the healthcare system. Currently, primary healthcare services do not align with the “felt needs” of the community. Practical recommendations to address the social, cultural, economic, and health system determinants include enabling and empowering people with diabetes and hypertension and their families to engage in self-management, improving existing health information systems, ensuring the availability of diagnostics and first-line drug therapy for diabetes and hypertension, and encouraging the use of single-pill combination (SPC) medications to reduce pill burden. Ensuring equitable access to drugs may improve hypertension and diabetes control in most disadvantaged groups. Furthermore, a more comprehensive approach to healthcare policy that recognizes the interconnectedness of non-communicable diseases (NCDs) and their social determinants is essential.

## 1 Introduction

Noncommunicable diseases (NCDs), also known as chronic diseases, are a major global health challenge responsible for more than two-thirds (73%) of all-cause mortality (1). Furthermore, the majority of NCD deaths occur in low- and middle-income countries (LMICs) (2). Importantly, many NCD-related deaths are premature and preventable (2, 3). Even after a decade since the launch of the National Programme for Prevention & Control of Non-Communicable Diseases (NP-NCD) by the government of India, the prevalence, awareness, treatment, and control rates of diabetes and hypertension remain abysmally poor in primary care settings with notable variations across districts, age, and socio-economic groups (4–6).

Globally, primary health care is recognized as the cornerstone of developing a resilient health system approach to preventing and controlling NCDs (7, 8). Previous evidence suggests that an effective primary health care system contributes to NCD control by encouraging a healthy lifestyle, preventing the onset of NCDs, decreasing premature NCD-related deaths, improving the quality of care, and reducing hospital admissions related to NCDs (9–11). Additionally, Primary Health Centers (PHC) fosters community involvement in NCD prevention and control, ensuring equitable NCD care access (12). However, in India, health systems were initially designed to address acute communicable diseases and maternal and child health (13) but now grapple with the challenge of delivering care for chronic conditions (14, 15). The primary care system for NCDs remains weak with underfunding, fragmented service delivery, and poorly functioning referral systems and faces significant resource constraints, including limited health workforce, medicine, supplies, and infrastructure (16–19). Further, this situation is worsened by disparities of socio-economic class, caste, and gender, which manifest as health inequalities (20). Recently, the Government of India launched the National Health Protection Mission known as the *Ayushman Bharat* to bring a comprehensive range of services closer to communities by transforming PHCs and sub-centers into Health and wellness centers (HWCs) and achieve universal health coverage in India (21–24).

In parallel, the Government of Kerala initiated the “*Aardram Mission*” in 2017 to revitalize the PHCs, recognizing the importance of strengthening universal primary care (25). This transformation involved upgrading PHCs to Family Health Centers (FHCs), which aligned with the national model of HWCs, with additional healthcare providers, and a comprehensive package of primary care services (25). Furthermore, the services at PHCs were strategically restructured to address the current epidemiological situation, with a particular focus on NCDs (26, 27). The increasing prevalence of diabetes and hypertension is considered a primary driver of this epidemiological transition.

Despite the discourse in policies regarding the importance of comprehensive care for managing diabetes and hypertension, research on patient experience remains under-investigated. Evidence highlights the central role of patient experience in healthcare quality (28). Therefore, mapping the entire patient journey and understanding various factors that influence how people with diabetes and hypertension access the healthcare system when needed is the key to understanding the patient experience and identifying any existing gaps. Against this background, our study aimed to explore the experiences of persons living with diabetes and hypertension (PLWDH) regarding their access to primary health care services to manage these conditions and to examine if the community expectations from FHCs resonate with the above-proposed policy direction.

## 2 Methods

### 2.1 Study design

This study is an exploratory (29) descriptive (30) qualitative research involving data collection through semi-structured interviews and focus-group discussions conducted among persons with diabetes, hypertension, or both in outpatient settings in Malappuram, Kerala.

### 2.2 Study setting

We conducted this study in purposively selected four FHCs of Malappuram, Kerala, from September 2022 to March 2023. Notably, all these health centers underwent a transformation in the initial phase of the *Aardram* mission in 2017. Malappuram, a northern district in Kerala, has a population of 4,112,920 (31). Malappuram was among the first districts in Kerala where the National Program for NCDs was implemented, and the community-based palliative care model for NCD was piloted in 1996 (32). A recent study in the district reported an NCD-multimorbidity prevalence of 39.8% among people seeking care from FHCs. Notably, the prevalence was higher in men (42.6%) than in women (38.1%). Hypertension and diabetes were the predominant coexisting chronic conditions (33). Malappuram is divided into 15 health blocks, with 59 fully functional FHCs implementing the national program for preventing and controlling non-communicable diseases (NP-NCD) across these blocks (33).

FHCs provide a range of services--- including health promotion activities, screening, laboratory services, and outpatient management for patients with diabetes and hypertension under the NCD program. The state has recently initiated the population-based screening of NCDs and risk factors (26), facilitated by Accredited Social Health Activists (ASHAs) and (JPHN), and suspected cases are referred to medical officers at FHCs. Patients requiring specialized care are referred to NCD clinics in community health centers or district hospitals. Additionally, the program offers free medicines for diabetes and hypertension at these facilities, with patients receiving their prescribed medications every month following consultation at the FHCs. Electronic health records and information systems at the patient level are being introduced in these centers (34).

### 2.3 Study participants, sampling strategy

The study population included adult individuals living with confirmed diabetes/hypertension or both, regardless of the disease stage or control status, in four FHCs in the Malappuram district. Purposive sampling (35) was used to identify eligible participants, following inclusion criteria: adults (18 years of age and older) seeking healthcare from public facilities or both public and private healthcare facilities for a minimum of six months. The assistance of local community health workers facilitated the identification of participants.

### 2.4 Data collection

In the first phase, semi-structured interviews were conducted face-to-face (36) with twenty adult participants. RR conducted all interviews in Malayalam using topic guides (Supplementary material S1). Pre-testing of the topic guides was undertaken before data collection. Each selected respondent was interviewed in a calm setting within health facility premises. All interviews were audio-recorded, and their duration ranged from 20 to 60 minutes.

We conducted one focus group discussion (FGD) (37) with six adults in the study area during the second phase. A heterogeneous group was selected for the FGD with different control statuses – achieved, not achieved, and taking insulin/oral hypoglycaemics or both, diagnosed but did not initiate the treatment. An experienced social worker and a junior public health nurse identified and recruited eligible participants for FGD. A topic guide was used to introduce different themes during the discussion. Basic demographic details like age, gender, marital status, education, and occupation were collected from each participant. Topics encompassed patients’ experiences of living with a chronic illness, health care utilization, and challenges in accessing health care. The FGD was audio-recorded and lasted for 60 minutes.

RR translated and transcribed all the interviews. Transcripts were checked multiple times to minimize mistakes made during transcription. Participant recruitment concluded following thematic saturation (meaning the researchers collectively determined that additional interviews were unlikely to yield new insights or information on the subject) (38). In total, 26 people participated in the study.

### 2.5 Data analysis

The analysis of interview transcripts and fieldwork notes employed a thematic analysis approach (39). A coding framework was developed after multiple readings of the transcripts and notes (40). This framework was systematically applied to the dataset with attention to recurring codes and their clustering around specific contexts. The coded data were organized to facilitate thematic interpretation. Ongoing discussions among all authors informed this analytical process, with consensus achieved through iterative reviews of codes and themes. Emphasis was placed on discerning similarities and differences across participants’ data, aiming to construct an explanation of the findings. Notably, any deviant cases were scrutinized to enhance the trustworthiness and rigor of the qualitative analysis (41).

The thematic framework derived from this analytical process closely aligns with the conceptual framework proposed, which focuses on the patient’s ability to access healthcare services (42). However, it is important to note that this framework was not applied as an *a priori* model for the research; instead, it was introduced in a subsequent stage to inform further analysis of patients’ health-seeking pathways and to explore perceived barriers to care among these healthcare users. The Levesque framework encompasses different stages of healthcare-seeking pathways, providing a valuable lens for examining the narratives of the study participants.

**Figure 1:**
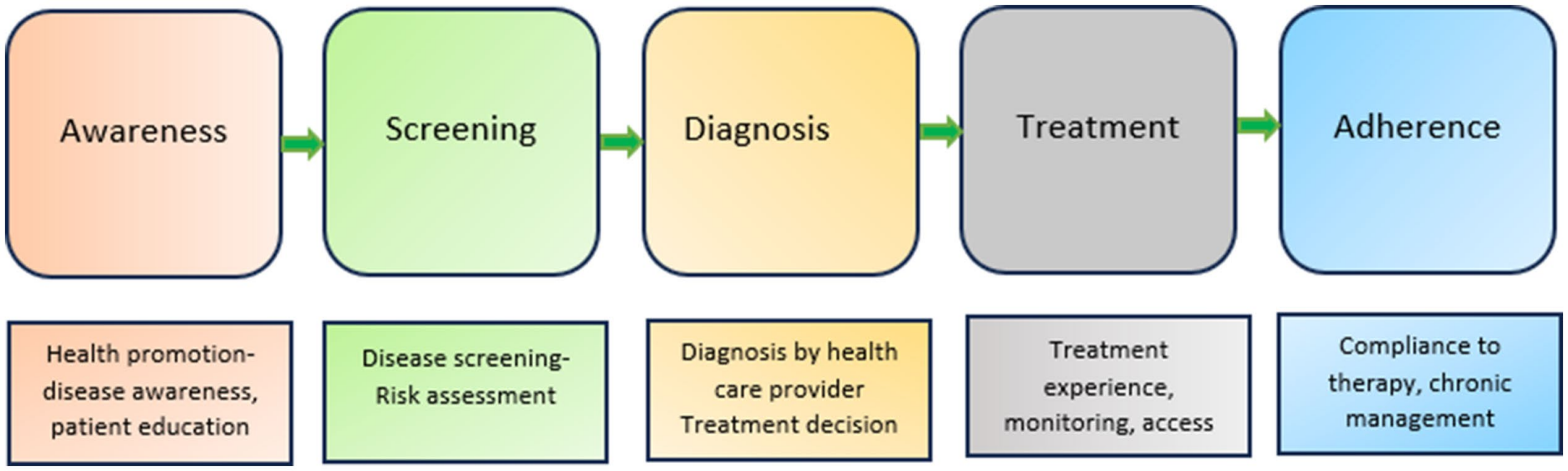
Patient journey for NCDs [Reprinted from Devi et al. (43)].

The patient journey for NCDs can be structured into five stages: awareness, screening, diagnosis, treatment, and adherence (Figure 1) incorporating rehabilitation (43). The accessibility of services shapes the patient’s journey. Levesque et al.’s access-to-care model served as our conceptual framework to examine the factors influencing access to care at both the health system and population levels. The model examined healthcare access across five dimensions: approachability, acceptability, availability and accommodation, affordability, and appropriateness. The above-mentioned access dimensions are influenced by the patient’s ability to perceive, seek, reach, pay, and engage. Access results from the interplay between supply and demand dimensions within the continuum of care, spanning from individuals’ recognition of health needs to healthcare outcomes (42).

## 3 Results

A total of 26 people living with diabetes/hypertension or both participated in the study. These participants varied in age, chronic conditions, control status, and background. The patients were aged between 43 and 67 years, with seven participants diagnosed only with diabetes, five with hypertension, and three with both diabetes and hypertension, while the remaining four participants suffered from other comorbidities associated with these conditions (Table 1). Most of them were unemployed (*n* = 14), and identified themselves as poor.

**Table 1:** Characteristics of study participants.

Participant ID	Age (in years)	Gender	Marital status	Level of Education	Occupation	Chronic NCD (self-reported diagnosis)
1	67	Female	Married	Lower Primary	Housewife	Diabetes hypertension
2	55	Male	Married	High school	Skilled worker	Hypertension
3	48	Male	Married	Completed high school	Technical worker	Diabetes, hypertension
4	52	Female	Married	Completed high school	Housewife	Diabetes
5	57	Female	Married	High school	Skilled worker	Diabetes
6	64	Female	Married	Lower Primary	Housewife	Diabetes
7	59	Female	Married	Secondary	Housewife	Diabetes, Hypertension, and Cardiovascular diseases (CVD)
8	45	Female	Married	Completed Secondary	Skilled worker	Diabetes
9	51	Female	Married	High school	Skilled laborer	Hypertension, asthma
10	63	Male	Married	Upper Primary	Manual laborer	Hypertension
11	44	Female	Widowed	Degree	Skilled worker	Hypertension
12	53	Female	Married	Completed high school	Skilled worker	Diabetes, hypertension
13	59	Female	Married	Completed secondary	Housewife	Hypertension
14	45	Female	Married	Completed Secondary	Technical worker	Diabetes
15	62	Male	Married	Lower Primary	Manual laborer	Diabetes, hypertension
16	56	Female	Married	Lower Primary	Unable to work	Diabetes, Hypertension, and CVD
17	66	Female	Married	Lower Primary	Housewife	Hypertension
18	56	Female	Married	Upper Primary	Housewife	Diabetes
19	48	Female	Unmarried	Secondary	Manual laborer	Diabetes
20	55	Male	Married	Lower Primary	Manual laborer	Diabetes
21	60	Female	Married	Upper Primary	Housewife	Diabetes and early-stage renal disease
22	66	Female	Married	Lower Primary	Unable to work	Diabetes
23	57	Female	Married	Complete Secondary	Housewife	Hypertension
24	43	Male	Married	Degree	Skilled worker	Diabetes and hypertension
25	51	Female	Widowed	Secondary	Housewife	Diabetes
26	48	Female	Married	Completed Secondary	Housewife	Hypertension

**Figure 2:**
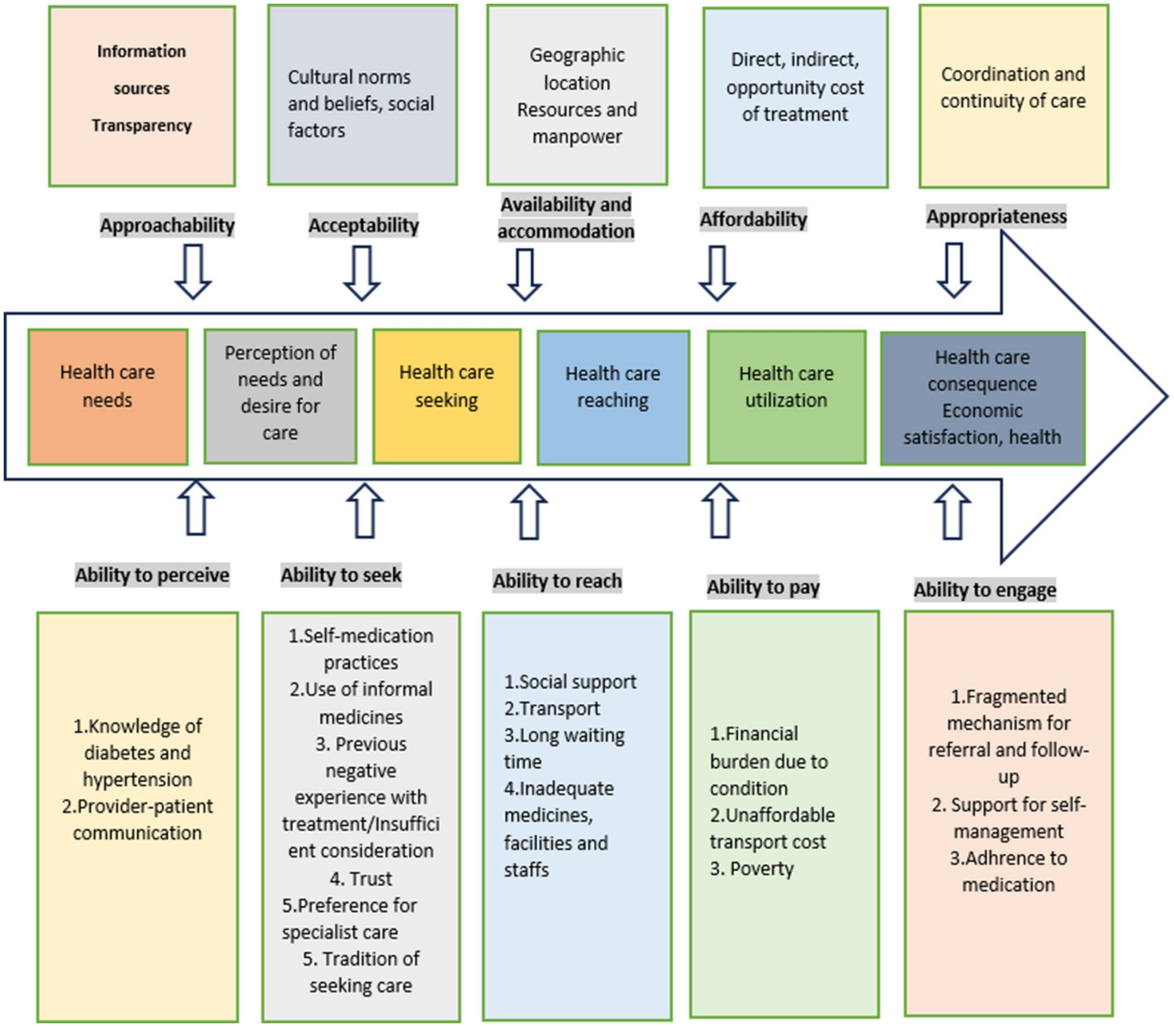
Conceptualization of access to care through patient perspectives from Kerala, India.

Our analysis demonstrated factors contributing to a complex health-seeking pathway for individuals with diabetes and hypertension. These factors align with the five domains outlined in the Levesque framework (42). The five identified themes correspond to the key access dimensions and associated abilities from the patient’s viewpoint, as depicted in Figure 2. The themes and the illustrative quotes are presented in Table 2. Additional quotes can be found in Supplementary Table S2.

**Table 2:** Themes and illustrative quotes.

Themes	Illustrative quotes
Approachability and ability to perceive	“Any health issue we have, I always call the JPHN* sister; I have called her even at 10 PM, and she answers all my queries very politely. She always explains in detail, and I can understand her. When she comes to Anganwadi, she always informs us about all activities and services in the health center. I shifted my treatment to a primary health center from a private one because she asked me. She knows my situation. In primary health centers, medicines, and services are free of cost, but in private, we should wait hours for tokens and pay big money.”**(44-year-old woman with diabetes)** *“I waited a year and a half to start medication after being diagnosed because I did not experience any issues related to this condition. There were no headaches, tiredness, or shivering. Shouldn’t we be concerned when these symptoms appear?.”**(59-year-old woman with hypertension)*** *“Following Ramadan, I experienced weight loss, increased water intake, and frequent urination. Unaware of these as diabetes symptoms, I was pleased with my weight loss and attributed frequent urination to staying well-hydrated. Nevertheless, friends and relatives noticed the weight loss, questioning its cause, and a relative suggested a blood test. The private lab reported a fasting sugar level above 230, indicating a high likelihood of diabetes, and advised an immediate doctor’s visit.”**(55-year-old woman with diabetes)*** *“Occasionally, I checked my blood pressure and blood sugar during the monthly NCD screening camp in Anganwadi. Both readings were slightly elevated, around 10–20 points higher than normal. The JPHN sister recommended diet restrictions in one visit for a potential cure and never asked me to consult a doctor or start medication. I tried to follow a restricted diet for 1.5 years and assumed I was all right. However, I faced severe pneumonia six months ago, impacting my lungs. I was hospitalized for over ten days at a private hospital. Tests revealed a blood sugar level above 350, affecting my kidneys and eyes. Since then, I’ve consistently taken medications for blood pressure, diabetes, and kidney concerns. The doctor stressed the lifelong need for medication.”**(60-year-old woman with diabetes and early renal disease)***
Acceptability and ability to seek.	*“I believe a comprehensive full body checkup and review are necessary due to my use of insulin, aspirin, and various medications for conditions like high blood pressure, high cholesterol, and diabetes. This is especially important after my heart surgery three years ago. Unfortunately, the primary health center lacks the required specialists and facilities for this evaluation.”**(56-year-old woman with diabetes, hypertension, and CVD).*** *“The hospital staff has designated treating people with diabetes or hypertension to Mondays and Wednesdays from the FHC. If we cannot make it on those days, the pharmacist reluctantly provides medicines, with grumbling. I once asked him why he was here if he could not accommodate other days. Our schedules do not always align with these days, as we have other commitments. When I raised this with a doctor, no action was taken. Respect for older adults should be a mutual value from childhood. I’m unhappy with staff behavior, but due to financial constraints, we have no choice but to return for medicines.”**(66-year-old woman with hypertension)*** *“In public healthcare facilities, the doctors often rush through appointments, barely allowing patients a moment to sit. They promptly reach for our notebooks and jot down medicines without inquiring about our concerns. Even when we try to communicate, they aren’t truly listening. There seems to be no need for them to refer to previous prescriptions nowadays. If we present our notebooks, they repeat the same medications. When consulted privately, the same doctor took the time to explain everything in detail to me.”**(44-year-old woman with hypertension)*** *“I heard from fellow visitors of this facility that the medicines offered here are of low dose and quality. I sought care from Dr. X at XX Hospital, a private establishment. Over time, I have consistently consulted him for various health concerns due to my trust in his expertise and prescribed medications. Upon reviewing all my medical reports, Dr. X assured me everything was normal. He said I was fit and healthy for my age, offering a reassuring pat on my shoulder. This brought about a sense of relief in me.”**(66-year-old woman with diabetes)*** *“I was taking allopathic medicines, and my sugar was in control, but then you see, people started telling me, that continuously taking allopathic medicines is dangerous, damaging my kidneys, so I was worried and thought of trying Ayurveda. I experimented with ‘kanthari Mallika kashayam,’ a herbal decoction, for a period. A traditional healer from Attapadi supplied the medicines monthly to my maternal home. In March, I consumed two bottles. After breakfast, the healer advised a daily intake of 200 milliliters in a glass of hot water. My relatives, including my uncle and brother, also used it and suggested it to me. Given our family’s strong history of diabetes—most family members over 45 years old have it—I decided to try it for a few months. I reduced my intake of other medicines and insulin, only taking half a tablet. However, I did not experience any benefits; I felt worse. Consequently, I resumed my allopathic tablets.”**(56-year-old woman with diabetes, hypertension, and CVD)***
Availability and ability to reach	*“Getting to XXX health center poses a significant challenge due to the absence of a suitable bus route. The limited daily bus schedule of 3–4 trips does not extend to the facility. Moreover, there is an uphill walk from the bus stop to the hospital premises. I feel tired to walk. I cannot cover the expense of taking an auto-rickshaw.”**(62-year-old man with diabetes and hypertension)*** *“Initially, I used to accompany two others (neighbors) on our visits, but since their blood sugar levels did not improve and they discontinued their visits, I found it difficult to continue going alone. Consequently, I have stopped visiting the facility temporarily.”**(66-year-old woman with diabetes)*** *“While improvements like infrastructure such as airport chairs and water facilities have been made, our most critical needs are still unaddressed. The efforts are commendable, but it would be highly beneficial if we could receive all necessary medications free of charge. We are willing to patiently wait in line if it means accessing medicines without cost.”**(FGD)*** *“From my experience, I have noticed that when I consume tablets available at health centers, there is no improvement in sugar levels; they remain persistently high and result in increased fatigue. However, it is only when I use insulin I observe a reduction. But I do not receive a consistent supply of three insulin bottles monthly.”**(60-year-old woman with diabetes and early renal disease)*** *“Doctor opts for fasting sugar tests, which lead me to private laboratories. The public health facility is often crowded, and enduring lengthy waits without eating becomes challenging. Typically, the lab procedures commence around 9 AM in this health center, which further adds to the difficulty of waiting without food.”**(45-year-old woman with diabetes).***
Affordability and ability to pay	*“Formerly, we received four bottles of insulin from this facility. However, this supply has ceased and we receive only two. Upon my inquiry to doctors, we were informed that medication is now prioritized for emergency and new diabetic cases. All of us are above 60 years old and unemployed, so purchasing medicines from private pharmacies is challenging. We are buying from Jan Aushadhi stores and spending 300 rupees per month to cover our medication expenses.”* **(*62-year-man with diabetes and hypertension)*** *“Everything’s expensive now. Sadly, I am also not receiving the old age pension, leaving me without financial support. I rely on the ration shop’s yellow card for free rice. Due to excruciating leg pain, I cannot work and even struggle to walk to the restroom. Seeking private medical consultation requires around 300–400 rupees, which I cannot afford. In government healthcare, I do not receive all the necessary medications.”**(62-year-old woman with diabetes and peripheral artery disease, for 12 years)*** *“I sought medical advice from Dr S a few years back, but I had to discontinue due to financial issues. Instead of visiting the doctor when I feel unwell, I occasionally purchase medicines using my previous prescription. However, my irregularity in seeking medical care has made me hesitant to return to the same doctor. The lack of money has caused me to lapse in my treatment. I am aware that diabetes is a chronic condition that requires consistent medication (pauses and tears up).”* ***(51-year-old woman with diabetes)***
Appropriateness and ability to engage	*“Occasionally, I experience headaches and a general feeling of unease, and I’m uncertain how to handle these situations. I usually opt to wait and observe if the symptoms alleviate naturally. If they persist, I may need to consider visiting the doctor at the FHC once more. Managing these health concerns comes with associated costs, such as transportation and medication expenses. So, I tolerate.”**(49-year-old woman with diabetes)*** *“In my perspective, when a patient visits a doctor or nurse, their soothing and comforting words alone can alleviate a significant portion of our distress. For instance, addressing us affectionately and engaging in casual conversation while administering medication can help dull the pain. When my husband was hospitalized at MMM (a private hospital), I vividly remember the kidney specialist who attended to him. This doctor would call and inquire, “How’s Appu Etta doing? What did you have for breakfast?” Those simple inquiries brought about a sense of ease and positivity. However, the doctor at the health center has a very quick temper and frequently raises his voice, making it difficult for us to approach them with any questions or concerns. He maintains a strict demeanor, and I fear him. Our interactions usually conclude with him prescribing medications, which we then purchase before returning home. I distinctly recall an incident where he scolded a diabetic person who wished to collect medications for his wife as well.”**(60-year-old woman with diabetes on insulin)*** *“When my leg pain worsens, I visit a private healthcare facility. However, during my visits, doctors scolded me for using medications for diabetes from public healthcare providers. There is the Jan Aushadhi store pharmacy, where medicines are cheaper. Private doctors allege that the public health centers and Jan Aushadi store distribute outdated medications, advising us to dispose of these tablets. They even warn that using such medicines might lead to other health issues. Some professionals go so far as to claim that any medication obtained for free is of subpar quality. As a result, I find myself uncertain where to place my trust.”**(62-year-old woman with diabetes and peripheral artery disease, for 12 years, on a woman with oral hypoglycemic)*** *“Many of us, particularly those dealing with diabetes and hypertension, share this general sentiment that the medicines provided at this facility are outdated and seem ineffective, which is the cause behind our uncontrolled blood sugar levels. During our wait to see the doctor, I’ve heard numerous individuals express similar concerns about the medications.”* ***(FGD)*** *“When I feel excessively tired, I may take a whole pill instead of the usual half. Generally, there is not much else that needs to be done. Doctors typically continue prescribing the same medications. As a result, I do not find it necessary to make monthly visits to the clinic.”**(55-year-old man with diabetes)*** *“I try to include salads or opt for wheat-based foods during dinner. I have learned about dietary limitations and the significance of physical activities such as walking and sit-ups through sessions and gatherings held at the government hospital in Perinthalmanna. In the past, I used to engage in walks lasting 1 to 1.5 h, often with the company of my friends. Unfortunately, following my heart surgery, I’ve had to discontinue all forms of physical activity due to my limitations. Despite this, I still manage to carry out routine tasks at a more leisurely pace.”**(56-year-old woman with diabetes, hypertension, and CVD, underwent surgery three years ago)*** *“I am consuming a considerable number of tablets each day, three before meals, four after meals. Additionally, I administer insulin right after eating. Following this medication routine and accompanying it with water, my stomach feels quite full, making me lose my appetite for dinner?! This poses a significant concern for me, as extensive use of medicines leaves me wondering whether my body can tolerate too many medicines. If possible, I wish to curtail few medicines.”**(56-year-old woman with diabetes, hypertension, and CVD, underwent surgery three years ago)***

### 3.1 Approachability and ability to perceive

Approachability refers to the “ability of individuals with healthcare needs to identify, to seek information on the existing forms of services and perceive their potential impact on their health” (42). Some participants said they were provided sufficient information on the services available in health facilities through community health staff like ASHAs and JPHNs. Few participants recalled outreach screening activities for diabetes and hypertension conducted in public spaces like Anganwadi’s in their area. However, many patients also reported several other sources of information available to them besides those provided by community health workers and doctors. For instance, patients gained prior knowledge through personal experience from family members, friends, and media. Patients reported these sources provided information on lifestyle changes, medication adherence, etc.

Participants’ ability to perceive the need for care was determined by factors such as patients’ knowledge about health conditions (perception of risk), age, education, and beliefs related to health and illness. Ensuring timely access to healthcare services and optimal adherence to long-term treatments relied on patients’ understanding of their chronic condition when symptoms arise. Most of the patients reported being aware of hypertension and diabetes before diagnosis. However, participants reported sub-optimal knowledge of preventive measures and treatment needs. Older women interviewed in the study were unaware that hypertension is usually asymptomatic, and there was limited knowledge of potential outcomes. When asked what causes hypertension, one patient interviewed reported that stress causes hypertension. Some of the participants failed to understand their symptoms were indicative of diabetes and hypertension and that addressing their condition might entail more than just making lifestyle changes; instead, it may require long-term treatment as well. Some participants also believed that chronic diseases like diabetes and hypertension only impact older adults.

Instances were reported when patients did not receive sufficient information and support from the healthcare field staff to develop an adequate understanding of their condition, even after being suspected of diabetes or hypertension, in screening camps. Inadequate knowledge about their conditions led these participants to wait until their conditions were no longer manageable, leading to delays in seeking care.

In general, when assessing the patients’ views in our study on diabetes and hypertension, the younger individuals minimized its importance. In contrast, mostly the older group of patients acknowledged the severity of the condition and sought treatment.

### 3.2 Acceptability and ability to seek

This theme addresses “the social and cultural factors that affect patient’s ability to receive and accept healthcare services” (42). Our conversations showed that study participants desired FHCs to function as health facilities catering to minor health concerns. There was a general acknowledgment among people that FHCs were not equipped to handle complex medical conditions. As a result, people did not anticipate that FHC staff would address all health issues. Moreover, patients did not expect to get integrated care for managing multiple chronic conditions from FHCs.

Despite the nature of the visit being routine follow-ups, some patients preferred specialist services if their budget allowed. However, the availability of free medications and services and proximity to the residence enticed them to seek care from the FHCs.

We found that private care with specialists was predominantly reserved for managing immediate medical issues or complications arising from their conditions. The participants also commonly believed that higher-level hospitals would offer superior healthcare due to enhanced diagnostic capabilities and skilled medical professionals. Most of the patients had consulted multiple providers since their initial diagnosis.

The participants’ social background and trust in the healthcare system affected their ability to access healthcare services. The degree of trust was influenced by their personal (or social network’s) prior encounters with services, the reputation of selected health facilities, and the belief that higher-level health facilities offered better service quality. Prescribers’ attitudes influenced trust in treatment. People had preconceived notions about the doctors and other staff. People often complained that doctors in FHCs failed to give them sufficient attention and respect. The perception of the government doctors’ indifferent attitude stemmed from the belief that they were not incentivized or paid extra at government facilities. Patients also believed that in private practice, doctors spent time and prescribed medications that were more effective in improving blood sugar levels and controlling blood pressure, enhancing the perception of better healthcare outcomes under private care. Thus, in addition to expertise in clinical skills, patients valued the doctor’s communication style. On the other hand, we observed a decline in trust in a physician due to an unfavorable experience, leading a participant to abstain from using health services provided by that doctor.

Many participants were accustomed to pursuing alternative therapies, such as traditional medicines, rooted in their cultural practices. Our interviews with some patients revealed that they discontinued the prescribed allopathic medications and adopted traditional medicines without any formal advice after achieving the control status. Some patients considered medicines as chemicals with potential side effects and resorted to alternate systems of medicines. While others developed a fear of becoming dependent on medications lifelong, leading them to explore alternative treatments to reduce reliance on medicines provided by the facility. Previous lack of improvement with allopathic treatment and success stories from people who have experienced positive results with alternate treatment prompted a few to consider these options.

### 3.3 Availability and ability to reach

This theme concerns “the extent to which people can avail healthcare services” (42). Few participants highlighted that the remote geographic location of the health center hindered their access to healthcare services. Private transportation to healthcare facilities was expensive and challenging for older patients, restricted by the distance they could travel.

The capability of the older participants to reach the facility was influenced by the presence of a family member or neighbor who could accompany them to the healthcare facilities.

Most respondents emphasized that in the past few years (due to the restructuring of primary care services under the *Aardram* mission), they have experienced improved healthcare services through upgrades in infrastructure, greater availability of doctors and laboratory facilities, and the introduction of evening out patients’ departments (OPDs). While expanded provision of the services was positively viewed to enhance accessibility, respondents noted the major deficiencies in service adequacy. A prevailing concern among most patients was the irregular availability of free medicines, leading to challenges in adhering to their prescribed treatment. Several patients reported that the shortage of drugs compelled them to personally bear the cost of purchasing them from local pharmacies. Furthermore, few patients expressed dissatisfaction with the facility’s restricted range of treatment options, often limited to just two or three medications for addressing their medical conditions.

Patients who had multiple NCDs described intensified symptoms associated with a lack of access to medicines. One patient talked about the challenges she faced in accessing a reliable supply of insulin.

In some cases, health services were available to patients, but this did not translate to services being accessible to patients. For instance, in health centers, laboratories and pharmacies were available at the same location. Still, these services were often unavailable due to a lack of supplies, medicines, or human resources. Most participants said the public health facility was usually crowded, with substantial waiting times and an inconvenience in managing fasting tests due to the overcrowding and the timing of lab procedures. Consequently, many patients resorted to visiting private clinics or laboratories to monitor their glucose levels, incurring additional time and expenses. Almost all the patients voiced their dissatisfaction with being asked to return to FHC every month to obtain medicines, finding it inconvenient and burdensome.

### 3.4 Affordability and ability to pay

This theme encompasses the economic capacity of people to afford healthcare expenses. Despite medications being free at public health facilities, the recurring scarcity of medicines and the need for frequent doctor visits were deemed financially challenging for many. This financial barrier had two aspects: the expense of regular medications and the cost of immediate prescriptions during symptom exacerbations, requiring hospitalizations, antibiotics, etc. Participants perceived that private health facilities offer superior service quality, leading many participants to choose out-of-pocket expenses for these hospitals despite the financial strain. Most respondents reported they lacked any form of health insurance.

The burden of out-of-pocket spending was highlighted as a core issue for older adults with chronic illnesses. Out-of-pocket expenses and financial constraints resulted in treatment gaps and often resulted in making choices about which medications to buy following a doctor’s prescription.

Participants emphasized that time spent on all-day travel hindered patients from working, earning an income, or attending to family responsibilities. Young male patients reported discontinuing monthly visits to public facility doctors due to the substantial time investment.

### 3.5 Appropriateness and ability to engage

Appropriateness refers to “how well services align with client requirements, including timely delivery, a thorough evaluation of health issues for accurate treatment determination, and the technical and interpersonal excellence of services” (42).

The long-term management of diabetes and hypertension demands consistent adherence to a treatment plan. Various factors affected the participants’ ability to follow their extended management plan. Long-term care involved regular hospital visits for obtaining medications and during worsening of symptoms. However, not all patients in our study could manage regular visits due to affordability issues, which interfered with adherence. The patient-provider relationship was an important factor related to adherence and patient satisfaction. Our interviews with patients revealed trust was is fostered when providers see patients as individuals, not merely cases. Additionally, displaying acceptance and respect contributed to nurturing this sense of confidence. Patients also expressed their frustration while reporting that they often left OPDs without getting the information they wanted, feeling overwhelmed and unable to understand the information provided. It was evident from patients’ accounts that they lacked preparedness to manage their health conditions when experiencing symptoms like pain and fatigue. These factors contributed to feelings of uncertainty about their future health. However, a few patients felt capable and confident in managing their conditions. They acquired skills through diverse experiences—some from their encounters and others from both within and outside the healthcare system. Patients with positive experiences in managing their conditions exhibited greater motivation to remain committed to long-term care.

People with multiple chronic conditions often required multiple healthcare providers, which were rarely coordinated with one another. Most of the interviewed patients with multimorbidity reported visiting different doctors and usually received conflicting advice. A patient in our study felt conflicted because private doctors criticized her for using medications from government healthcare providers. Private doctors claimed these medicines might be outdated or of poor quality. This created confusion and uncertainty for her when deciding whom to trust for her healthcare. Furthermore, some patients during discussions shared their belief that any medicines received for free could be of lower quality.

Most patients believed that their medical conditions were both progressive and permanent, with little influence from either themselves or their doctors on their future prognosis. This perspective made it difficult for them to grasp the concept of preventive care and how it could benefit them. Patients adhered to the advice they perceived as beneficial, pragmatic, and able to sustain them. Some patients mentioned that they were more inclined to follow medication prescriptions at the time of diagnosis. However, as they gained more experience and knowledge, they became discerning about the advice they followed and adjusted it to fit their situations. Interestingly, patients had received dietary advice from various sources outside the health center, with the most common sources being private doctors, community members, and social media.

Patients dealing with multiple health conditions in our study emphasized the influence of pill burden on their adherence to therapy. They found it challenging to cope with a substantial number of daily tablets. Moreover, the quantity of tablets also appeared to affect how patients subjectively viewed their health status.

## 4 Discussion

This study provides valuable insights into the healthcare processes of people living with diabetes and hypertension in public health facilities in Kerala, India. The access to Healthcare conceptual framework (42) enabled us to analyze the intricate process of access within the health systems and the context of the population. Our analysis identified various factors that influence the access to primary health services for the management of diabetes and hypertension, including participants’ limited knowledge of their condition, self-medication practices, physical distance to health facilities, availability of social support, high out-of-pocket expenditure, lack of trust, attitude of healthcare providers, medicine availability, and pill burden. Currently, primary healthcare services often do not sufficiently address the community’s needs. Provision of care to individuals with diabetes and hypertension was often fragmented, leading to multiple visits to advanced healthcare facilities (hospitals), consultations with specialists, and the simultaneous use of various medications. These findings are similar to a study in the Kolar district of Karnataka, which found that the fragmented “treatment-in-silos” hindered patients’ long-term adherence to treatment, especially as they age or face disabilities (19).

In our study, participant’s understanding of diabetes and hypertension varied and was often linked to their symptoms, presence of comorbidities, age, and education. The practice of self-medication emerged as a prevalent health behavior, where people sought advice from relatives or friends and obtained traditional remedies. Formal allopathic healthcare was mostly sought when self-medication did not improve their condition, potentially delaying timely treatment and sometimes even worsening the condition. Diagnosed patients also practiced self-medication when their symptoms were controlled. Previous studies reported using self-medication, including traditional herbal remedies, in Indian healthcare (44, 45). During the interviews, participants also spoke about the restricted variety of drugs at PHCs and frequently expressed doubts about the effectiveness of these drugs for their conditions.

Participants in our study who were predominantly from a low economic status reported limited access to hypertension and diabetes services. Previous evidence suggests clustering of NCDs in disadvantaged groups who have low education status and live in poor communities. These, together with inadequate access to health care, further exacerbate health inequalities in NCD (46). The *Makkale Thedi Maruthuvam*” scheme is an initiative by the Government of Tamil Nadu to enhance equitable access by screening people aged 30 years and above for diabetes and hypertension, delivering essential medicines, and reviewing treatment compliance at the doorsteps of eligible patients (47). Such initiatives may reduce the frequency of monthly visits for medication refills and improve follow-up care. Practical actions to reduce NCD inequities should encompass prioritizing the service delivery and follow-up for the most susceptible people with diabetes and hypertension based on risk assessment and disparities in access to care.

Access to care was facilitated through support from family and other social networks. This finding is consistent with earlier research, as systematic reviews have established that social support enhances treatment adherence among individuals with NCDs by offering consistent reminders for timely medication consumption (48–50). In high-income countries, well-designed randomized control trials have demonstrated that diabetic populations benefit from peer/CHWs support, improving clinical and behavioral outcomes, including better glycaemic control (51–53). Programs like the UK’s Expert Patient Program provide a cost-effective approach combining peer support and structured education to enhance chronic health support (54, 55). Similarly, the Kerala Diabetes Prevention Program (K-DPP) (56) is a community-based peer-support lifestyle intervention program adapted from evidence-based interventions developed initially in high-income countries and has successfully reduced cardiometabolic risk in high-risk adults over two years. Furthermore, K-DPP encouraged healthier behaviors in peer leaders, like increased physical activity, improved dietary habits, and reduced alcohol consumption (57).

Participants recognized hypertension and diabetes as a lifelong disease, but older individuals were more committed to medication adherence. However, their understanding of long-term prevention steps and self-management was limited due to inadequate communication and support from healthcare workers at all healthcare system levels. Many patients felt overwhelmed by the complexity of managing chronic disease, struggling to initiate and sustain health-promoting behaviors due to uncertainty about where to start. In previous studies most patients seeking care from public healthcare facilities reported minimal or no assistance for self-management (19, 58). This highlights the need to enable and empower patients to participate in managing their care along the continuum spanning from prevention to end of life. Self-care or self-management is one form of intervention that has been of particular interest to manage chronic conditions better and address the socioeconomic challenges LMICs face (59). Many experts have asserted that effective management of chronic diseases occurs when patients are empowered to participate in their care actively. Most chronic care models, including the chronic care model, support self-management skills as an essential component of chronic care (60). Self-management interventions, including SMS-based education, guideline-based teaching sessions, and telephonic counseling, have demonstrated the potential to improve patient health outcomes (61–63). Our findings indicate the need for further research on self-management strategies for CVD, focusing on long-term sustainability, to suit the Kerala context.

Participants’ access to healthcare services was strongly shaped by their trust in the health system and social environment. Trust was influenced by previous personal or network experiences, the reputation of healthcare facilities, and the belief that higher-level facilities offer better service quality and prescribers’ attitudes. While FHC doctors reported to address patients hastily, people expected extensive conversations and meticulous examinations. This finding aligns with previous research, emphasizing the significance of positive patient-health professional relationships on patients’ adherence to treatment (64), particularly when patients perceive healthcare providers as compassionate and caring.

Among our study participants, medication non-adherence stemmed from multifaceted factors, encompassing psychosocial elements (perceived lack of social support), structural challenges (clinic distance and medication expenses), therapy-related issues (multiple visits, polypharmacy, pill burden), and barriers within the healthcare system (e.g., insufficient counseling, erratic medicine supply from public health facilities, and patient-provider relationship). Additionally, patients trust in healthcare systems and beliefs regarding medications and their perceptions of their illness contribute to non-adherence. Evidence suggests that the use of single-pill combination (SPC) is safe and has the potential to improve patient adherence and blood pressure control, particularly in LMICs (65). In 2019, the World Health Organization (WHO) incorporated SPC into the Essential medicines list (66). We encourage the uptake and use of SPCs in primary care settings to improve blood pressure control and simplify treatment regimens.

Previous studies have proposed that an integrated approach to NCD care can streamline and save patient time and costs. For example, Peck et al. (67) have shown that integrating NCD and human immunodeficiency virus (HIV) treatment services reduces patient travel burden, allows personalized treatment approaches, and addresses the lack of support for patient follow-up and self-management in the current healthcare system. Frenk discusses integrating interventions and adopting a diagonal approach to health system improvements across multiple conditions while emphasizing the need for tangible improvements in health outcomes (68).

Our further observation pertains to the linear nature of Levesque et al.’s framework (42). While this framework has recognized factors influencing the healthcare-seeking pathways of individuals with NCDs, it does not fully explain why patients progress through these multidirectional pathways. Consistent with Nguyen et al.’s findings (69), we observed that patients frequently navigate the care pathway in a non-linear manner due to the challenges of diagnosis and the long-term management of chronic conditions. This process involves loops and cycles, with patients moving forward and backward in pursuit of healthcare across various facilities. Consequently, an alternative non-linear representation, incorporating broader healthcare system factors, would better depict the interconnected elements influencing access to healthcare services.

Consistent with prior research, our study reveals that individual “abilities” are primarily influenced by the “system” and its deficiencies, rather than solely being the responsibility of individuals. Thus, the central aspect of any intervention may involve a substantial investment in enhancing the accessibility of the system to empower an individual’s “ability” to interact with it effectively.

### 4.1 Strengths and limitations of the study

The study has several limitations. Firstly, in its emphasis on capturing patients’ viewpoints, the study did not incorporate the healthcare workers (supply side), which could have provided valuable insights. Secondly, although efforts were made to include equal representation of male and female participants, most respondents were women, reflecting the predominant population seeking healthcare in the state. Thirdly, the quality of care provided to the patients and the resulting health outcomes were beyond the scope of the study.

A key strength of our study is that we have considered the most prevalent NCDs, like diabetes and hypertension, to explore how patients seek care for chronic conditions in Malappuram. Although variations may exist among different illnesses, experiences are likely similar due to the current state of the healthcare system in Kerala and India. Primary care services are weak, and health systems lack capacity to address chronic illnesses. Secondly, our prolonged engagement within the study sites, as we regularly visited the facilities and stayed in the local community, enabled us to obtain data with rich insights into how people experience care. Thirdly, using Levesque’s access framework in the analysis enhances the potential for replication of this study in other contextually similar settings.

### 4.2 Implication for practice and research

Preventing the rising burden of NCDs is challenging as it necessitates addressing the underlying social determinants of health (70). Moreover, the organization of care for individuals with NCDs is intricate and resource-intensive, and multiple concurrent chronic conditions, known as multimorbidity, further complicate matters (71–74). It is important to recognize that the roots of NCDs are deeply entrenched in society’s social, political, and economic structures.

Unfortunately, it appears that those shaping current policies and resource allocation are either unaware of or choose to overlook the realities on the frontlines of healthcare. Physicians often find themselves bridging the gap between treating individual illnesses and meeting the needs of patients grappling with multimorbid NCDs. Reachable and reliable care and support must be ensured at the initial point of contact. Healthcare professionals must deliver attentive support tailored to individuals’ perceived needs, incorporate self-management support to influence patients’ behavior, and maximize the use and effectiveness of community service. Furthermore, we need a more comprehensive approach to healthcare policy that recognizes the interconnectedness of NCDs and the social determinants that affect their occurrence and management. Such a shift in policy and resource allocation is essential to effectively address our society’s growing burden of NCDs.

## 5 Conclusion

The patient journey in managing diabetes and hypertension is multifaceted, involving multiple entry and exit points within the healthcare system. This study underscores the complexity of healthcare access and how access was affected by various factors. Unfortunately, there is a lack of a patient-centred care process that delves into the self-management efforts required by patients and explores how healthcare providers can offer adequate support. Consequently, it is crucial to rethink the design and organization of care for individuals with chronic diseases. This involves reframing the patient journey, acknowledging it as one characterized by multiple touchpoints where patients may initiate their healthcare pathway at different stages but consistently require good quality, affordable care and support at various junctures.

## Data availability statement

The raw data supporting the conclusions of this article will be made available by the authors, without undue reservation.

## Ethics statement

The studies involving humans were approved by Institutional Ethics Committee of Sree Chitra Tirunal Institute for Medical Sciences and Technology (SCT/IEC/ 1908). The studies were conducted in accordance with the local legislation and institutional requirements. The participants provided their written informed consent to participate in this study.

## Author contributions

RR: Conceptualization, Data curation, Formal analysis, Funding acquisition, Methodology, Writing – original draft, Writing – review & editing. PS: Conceptualization, Methodology, Supervision, Writing – review & editing. SS: Conceptualization, Methodology, Supervision, Writing – review & editing. KT: Conceptualization, Methodology, Supervision, Writing – review & editing. PJ: Conceptualization, Formal analysis, Funding acquisition, Methodology, Supervision, Writing – review & editing.

## References

[1] Lancet T. GBD 2017: a fragile world. Lancet. (2018) 392:1683. doi: 10.1016/S0140-6736(18)32858-7, PMID: 30415747

[2] Niessen LW Mohan D Akuoku JK Mirelman AJ Ahmed S Koehlmoos TP. Tackling socioeconomic inequalities and non-communicable diseases in low-income and middle-income countries under the sustainable development agenda. Lancet Lond Engl. (2018) 391:2036–46. doi: 10.1016/S0140-6736(18)30482-3, PMID: 29627160

[3] WHO. (2023) Non communicable diseases. Available at: https://www.who.int/news-room/fact-sheets/detail/noncommunicable-diseases

[4] Amarchand R Kulothungan V Krishnan A Mathur P. Hypertension treatment cascade in India: results from National Noncommunicable Disease Monitoring Survey. J Hum Hypertens. (2023) 37:394–404. doi: 10.1038/s41371-022-00692-y, PMID: 35513442 PMC10156594

[5] Prenissl J Jaacks LM Mohan V Manne-Goehler J Davies JI Awasthi A. Variation in health system performance for managing diabetes among states in India: a cross-sectional study of individuals aged 15 to 49 years. BMC Med. (2019) 17:92. doi: 10.1186/s12916-019-1325-6, PMID: 31084606 PMC6515628

[6] Sarma PS Sadanandan R Thulaseedharan JV Soman B Srinivasan K Varma RP. Prevalence of risk factors of non-communicable diseases in Kerala, India: results of a cross-sectional study. BMJ Open. (2019) 9:e027880. doi: 10.1136/bmjopen-2018-027880, PMID: 31712329 PMC6858196

[7] WHO. (2023) WHO package of essential noncommunicable (PEN) disease interventions for primary health care. Available at: https://www.who.int/publications-detail-redirect/9789240009226

[8] Demaio AR Nielsen KK Tersbøl BP Kallestrup P Meyrowitsch DW. Primary health care: a strategic framework for the prevention and control of chronic non-communicable disease. Glob Health Action. (2014) 7:24504. doi: 10.3402/gha.v7.24504, PMID: 25095779 PMC4122819

[9] Haque M Islam T Rahman NAA McKimm J Abdullah A Dhingra S. Strengthening primary health-care services to help prevent and control long-term (chronic) non-communicable diseases in low- and middle-income countries. Risk Manag Healthc Policy. (2020) 13:409–26. doi: 10.2147/RMHP.S239074, PMID: 32547272 PMC7244358

[10] Hansen J Groenewegen PP Boerma WGW Kringos DS. Living in A country with A strong primary care system is beneficial to people with chronic conditions. Health Aff (Millwood). (2015) 34:1531–7. doi: 10.1377/hlthaff.2015.0582, PMID: 26355055

[11] Bitton A Fifield J Ratcliffe H Karlage A Wang H Veillard JH. Primary healthcare system performance in low-income and middle-income countries: a scoping review of the evidence from 2010 to 2017. BMJ Glob Health. (2019) 4:e001551. doi: 10.1136/bmjgh-2019-001551, PMID: 31478028 PMC6703296

[12] Varghese C Nongkynrih B Onakpoya I McCall M Barkley S Collins TE. Better health and wellbeing for billion more people: integrating non-communicable diseases in primary care. BMJ. (2019) 364:l327. doi: 10.1136/bmj.l327 30692118 PMC6349006

[13] Vlassoff C Tanner M Weiss M Rao S. Putting people first: A primary health care success in rural India. Indian J Community Med. (2010) 35:326–30. doi: 10.4103/0970-0218.66896 20922117 PMC2940196

[14] Samb B Desai N Nishtar S Mendis S Bekedam H Wright A. Prevention and management of chronic disease: a litmus test for health-systems strengthening in low-income and middle-income countries. Lancet. (2010) 376:1785–97. doi: 10.1016/S0140-6736(10)61353-0, PMID: 21074253

[15] Gabert R Ng M Sogarwal R Bryant M Deepu RV McNellan CR. Identifying gaps in the continuum of care for hypertension and diabetes in two Indian communities. BMC Health Serv Res. (2017) 17:846. doi: 10.1186/s12913-017-2796-9, PMID: 29282052 PMC5746011

[16] Ramani S Sivakami M. Community perspectives on primary health centers in rural Maharashtra: what can we learn for policy? J Fam Med Prim Care. (2019) 8:2837–44. doi: 10.4103/jfmpc.jfmpc_650_19, PMID: 31681652 PMC6820439

[17] Sodani P Sharma K. Strengthening primary level health service delivery: lessons from a state in India. J Fam Med Prim Care. (2012) 1:127–31. doi: 10.4103/2249-4863.104983, PMID: 24479021 PMC3893968

[18] Bhaumik S. Health and beyond. Strategies for a better India: concept paper on primary health Care in India. J Fam Med Prim Care. (2014) 3:94–7. doi: 10.4103/2249-4863.137608 PMC4140008 25161962

[19] Lall D Engel N Devadasan N Horstman K Criel B. Challenges in primary care for diabetes and hypertension: an observational study of the Kolar district in rural India. BMC Health Serv Res. (2019) 19:44. doi: 10.1186/s12913-019-3876-9, PMID: 30658641 PMC6339380

[20] Patel V Mazumdar-Shaw K Kang G Das P Khanna T. Reimagining India’s health system: a Lancet citizens’ commission. Lancet Lond Engl. (2021) 397:1427–30. doi: 10.1016/S0140-6736(20)32174-7, PMID: 33308485 PMC9752001

[21] Angell BJ Prinja S Gupt A Jha V Jan S. The Ayushman Bharat Pradhan Mantri Jan Arogya Yojana and the path to universal health coverage in India: overcoming the challenges of stewardship and governance. PLoS Med. (2019) 16:e1002759. doi: 10.1371/journal.pmed.1002759, PMID: 30845199 PMC6405049

[22] Ministry of Health and Family Welfare, Governmnet of India. (2023) Operational guidelines on wellness interventions for Ayushman Bharat health and wellness Centres | National Health Systems Resource Centre. Available at: https://nhsrcindia.org/operational-guidelines-wellness-interventions-ayushman-bharat-health-and-wellness-centres

[23] Government Of India. (2022) Ayushman Bharat – national health protection mission. Available at: https://www.india.gov.in/spotlight/ayushman-bharat-national-health-protection-mission. (Accessed on September, 2022).

[24] Government Of India. (2022) Official website Ayushman Bharat. Available at: https://ab-hwc.nhp.gov.in/. (Accessed on January, 2022).

[25] Arogyakeralam. (2022) National Health Mission Kerala-Aardram. Available at: https://arogyakeralam.gov.in/2020/04/01/aardram/. (Accessed on October, 2022).

[26] Sadanandan R Sivaprasad S. Adding screening for “end organ damage” to the noncommunicable disease package in primary care. Indian J Ophthalmol. (2021) 69:3064–7. doi: 10.4103/ijo.IJO_1496_21, PMID: 34708743 PMC8725093

[27] Sivaprasad S Netuveli G Wittenberg R Khobragade R Sadanandan R Gopal B. Complex interventions to implement a diabetic retinopathy care pathway in the public health system in Kerala: the Nayanamritham study protocol. BMJ Open. (2021) 11:e040577. doi: 10.1136/bmjopen-2020-040577, PMID: 34183333 PMC8240569

[28] Oben P. Understanding the patient experience: A conceptual framework. J Patient Exp. (2020) 7:906–10. doi: 10.1177/2374373520951672, PMID: 33457518 PMC7786717

[29] Stebbins RA. Exploratory research in the social sciences [internet]. Thousand Oaks, CA: SAGE Publications, Inc (2001).

[30] Sandelowski M. What’s in a name? Qualitative description revisited. Res Nurs Health. (2010) 33:77–84. doi: 10.1002/nur.20362 20014004

[31] Population Census. (2022) Malappuram Municipality City population census 2011-2023 | Kerala. Available at: https://www.census2011.co.in/data/town/803269-malappuram-kerala.html. (Accessed on September, 2022).

[32] Philip RR Philip S Tripathy JP Manima A Venables E. Twenty years of home-based palliative care in Malappuram, Kerala, India: a descriptive study of patients and their care-givers. BMC Palliat Care. (2018) 17:26. doi: 10.1186/s12904-018-0278-4, PMID: 29444688 PMC5813368

[33] Ismail S Stanley A Jeemon P. Prevalence of multimorbidity and associated treatment burden in primary care settings in Kerala: a cross-sectional study in Malappuram District, Kerala, India. Wellcome Open Res. (2022) 7:67. doi: 10.12688/wellcomeopenres.17674.2, PMID: 35592547 PMC9086527

[34] Joseph L Greenfield S Manaseki-Holland S Lekha TR Sujakumari S Panniyammakal J. Patients’, carers’ and healthcare providers’ views of patient-held health records in Kerala, India: A qualitative exploratory study. Health Expect. (2023) 26:1081–95. doi: 10.1111/hex.13721, PMID: 36782391 PMC10154823

[35] Given LM. The SAGE encyclopedia of qualitative research methods. Thousand Oaks, CA: SAGE Publications (2008). 1073 p.

[36] Britten N. Qualitative interviews in medical research. BMJ. (1995) 311:251–3. doi: 10.1136/bmj.311.6999.251, PMID: 7627048 PMC2550292

[37] Caillaud S Flick U. Focus groups in triangulation contexts In: Barbour RS Morgan DL, editors. A new era in focus group research: Challenges, innovation and practice. London: Palgrave Macmillan UK (2017). 155–77.

[38] Hennink MM Kaiser BN Marconi VC. Code saturation versus meaning saturation: how many interviews are enough? Qual Health Res. (2017) 27:591–608. doi: 10.1177/1049732316665344, PMID: 27670770 PMC9359070

[39] Braun V Clarke V. Using thematic analysis in psychology. Qual Res Psychol. (2006) 3:77–101. doi: 10.1191/1478088706qp063oa

[40] Kiger ME Varpio L. Thematic analysis of qualitative data: AMEE guide no. 131. Med Teach. (2020) 42:846–54. doi: 10.1080/0142159X.2020.1755030, PMID: 32356468

[41] Pope C Ziebland S Mays N. Analysing qualitative data. BMJ. (2000) 320:114–6. doi: 10.1136/bmj.320.7227.114, PMID: 10625273 PMC1117368

[42] Levesque JF Harris MF Russell G. Patient-centred access to health care: conceptualising access at the interface of health systems and populations. Int J Equity Health. (2013) 12:18. doi: 10.1186/1475-9276-12-18, PMID: 23496984 PMC3610159

[43] Devi R Kanitkar K Narendhar R Sehmi K Subramaniam K. A narrative review of the patient journey through the Lens of non-communicable diseases in low- and middle-income countries. Adv Ther. (2020) 37:4808–30. doi: 10.1007/s12325-020-01519-3, PMID: 33052560 PMC7553852

[44] Vishnu N Mini GK Thankappan KR. Complementary and alternative medicine use by diabetes patients in Kerala, India. Glob Health Epidemiol Genomics. (2017) 2:e6. doi: 10.1017/gheg.2017.6, PMID: 29868217 PMC5870440

[45] Greenhalgh T. Drug prescription and self-medication in India: An exploratory survey. Soc Sci Med. (1987) 25:307–18. doi: 10.1016/0277-9536(87)90233-4, PMID: 3629304

[46] Cesare MD Khang YH Asaria P Blakely T Cowan MJ Farzadfar F. Inequalities in non-communicable diseases and effective responses. Lancet. (2013) 381:585–97. doi: 10.1016/S0140-6736(12)61851-0 23410608

[47] Thiagesan R Soundari H Kalpana B Gopichandran V. “Makkalai Thedi Maruthuvam” scheme in Tamil Nadu: an intersectionality-based analysis of access to NCD care. Indian. J Med Ethics. (2023) VIII:203–9. doi: 10.20529/IJME.2023.015 36945849

[48] Miller TA DiMatteo MR. Importance of family/social support and impact on adherence to diabetic therapy. Diabetes Metab Syndr Obes. (2013) 6:421–6. doi: 10.2147/DMSO.S36368, PMID: 24232691 PMC3825688

[49] Walsh JME McDonald KM Shojania KG Sundaram V Nayak S Lewis R. Quality improvement strategies for hypertension management: a systematic review. Med Care. (2006) 44:646–57. doi: 10.1097/01.mlr.0000220260.30768.32 16799359

[50] Glynn LG Murphy AW Smith SM Schroeder K Fahey T. Self-monitoring and other non-pharmacological interventions to improve the management of hypertension in primary care: a systematic review. Br J Gen Pract J R Coll Gen Pract. (2010) 60:e476–88. doi: 10.3399/bjgp10X544113, PMID: 21144192 PMC2991764

[51] Knowler WC Barrett-Connor E Fowler SE Hamman RF Lachin JM Walker EA. Reduction in the incidence of type 2 diabetes with lifestyle intervention or metformin. N Engl J Med. (2002) 346:393–403. doi: 10.1056/NEJMoa012512, PMID: 11832527 PMC1370926

[52] Pan XR Li GW Hu YH Wang JX Yang WY An ZX. Effects of diet and exercise in preventing NIDDM in people with impaired glucose tolerance: the Da Qing IGT and diabetes study. Diabetes Care. (1997) 20:537–44. doi: 10.2337/diacare.20.4.537, PMID: 9096977

[53] Tuomilehto J Lindström J Eriksson JG Valle TT Hämäläinen H Ilanne-Parikka P. Prevention of type 2 diabetes mellitus by changes in lifestyle among subjects with impaired glucose tolerance. N Engl J Med. (2001) 344:1343–50. doi: 10.1056/NEJM200105033441801 11333990

[54] Donaldson L. Expert patients usher in a new era of opportunity for the NHS: the expert patient programme will improve the length and quality of lives. BMJ. (2003) 326:1279–80. doi: 10.1136/bmj.326.7402.1279, PMID: 12805129 PMC1126164

[55] Tattersall R. The expert patient: a new approach to chronic disease management for the twenty-first century. Clin Med. (2002) 2:227–9. doi: 10.7861/clinmedicine.2-3-227, PMID: 12108472 PMC4954037

[56] Thankappan KR Sathish T Tapp RJ Shaw JE Lotfaliany M Wolfe R. A peer-support lifestyle intervention for preventing type 2 diabetes in India: A cluster-randomized controlled trial of the Kerala diabetes prevention program. PLoS Med. (2018) 15:e1002575. doi: 10.1371/journal.pmed.1002575, PMID: 29874236 PMC5991386

[57] Haregu T Aziz Z Cao Y Sathish T Thankappan KR Panniyammakal J. A peer support program results in greater health benefits for peer leaders than other participants: evidence from the Kerala diabetes prevention program. BMC Public Health. (2023) 23:1175. doi: 10.1186/s12889-023-16049-0, PMID: 37337201 PMC10278268

[58] Humphries C Jaganathan S Panniyammakal J Singh S Goenka S Dorairaj P. Investigating clinical handover and healthcare communication for outpatients with chronic disease in India: A mixed-methods study. PLoS One. (2018) 13:e0207511. doi: 10.1371/journal.pone.0207511, PMID: 30517130 PMC6281223

[59] Hearn J Ssinabulya I Schwartz JI Akiteng AR Ross HJ Cafazzo JA. Self-management of non-communicable diseases in low- and middle-income countries: A scoping review. PLoS One. (2019) 14:e0219141. doi: 10.1371/journal.pone.0219141, PMID: 31269070 PMC6608949

[60] Bodenheimer T Wagner EH Grumbach K. Improving primary Care for Patients with Chronic IllnessThe chronic care model, part 2. JAMA. (2002) 288:1909–14. doi: 10.1001/jama.288.15.1909 12377092

[61] Sherifali D Berard LD Gucciardi E MacDonald B MacNeill G. Self-management education and support. Can J Diabetes. (2018) 42:S36–41. doi: 10.1016/j.jcjd.2017.10.006 29650109

[62] McManus RJ Mant J Bray EP Holder R Jones MI Greenfield S. Telemonitoring and self-management in the control of hypertension (TASMINH2): a randomised controlled trial. Lancet. (2010) 376:163–72. doi: 10.1016/S0140-6736(10)60964-6, PMID: 20619448

[63] McLean G Band R Saunderson K Hanlon P Murray E Little P. Digital interventions to promote self-management in adults with hypertension systematic review and meta-analysis. J Hypertens. (2016) 34:600–12. doi: 10.1097/HJH.0000000000000859, PMID: 26845284 PMC4947544

[64] Mechanic D. Changing medical organization and the Erosion of trust. Milbank Q. (1996) 74:171–89. doi: 10.2307/3350245, PMID: 8632733

[65] Bruyn E Nguyen L Schutte AE Murphy A Perel P Webster R. Implementing single-pill combination therapy for hypertension: A scoping review of key health system requirements in 30 low- and middle-income countries. Glob Heart. (2022) 17:6. doi: 10.5334/gh.1087, PMID: 35174047 PMC8796691

[66] WHO. (2023) WHO model lists of essential medicines. Available at: https://www.who.int/groups/expert-committee-on-selection-and-use-of-essential-medicines/essential-medicines-lists

[67] Peck R Mghamba J Vanobberghen F Kavishe B Rugarabamu V Smeeth L. Preparedness of Tanzanian health facilities for outpatient primary care of hypertension and diabetes: a cross-sectional survey. Lancet Glob Health. (2014) 2:e285–92. doi: 10.1016/S2214-109X(14)70033-6, PMID: 24818084 PMC4013553

[68] Frenk J. Reinventing primary health care: the need for systems integration. Lancet. (2009) 374:170–3. doi: 10.1016/S0140-6736(09)60693-0, PMID: 19439350

[69] Nguyen TA Pham YN Doan NP Nguyen TH Do TT Van Vu G. Factors affecting healthcare pathways for chronic lung disease management in Vietnam: a qualitative study on patients’ perspectives. BMC Public Health. (2021) 21:1145. doi: 10.1186/s12889-021-11219-4, PMID: 34130687 PMC8207672

[70] Sreekumar S Ravindran TKS. A critique of the policy discourse on primary health care under the Aardram mission of Kerala. Health Policy Plan. (2023) 38:949–59. doi: 10.1093/heapol/czad041, PMID: 37354455

[71] Pefoyo AJK Bronskill SE Gruneir A Calzavara A Thavorn K Petrosyan Y. The increasing burden and complexity of multimorbidity. BMC Public Health. (2015) 15:415. doi: 10.1186/s12889-015-1733-2, PMID: 25903064 PMC4415224

[72] Mini GK Thankappan KR. Pattern, correlates and implications of non-communicable disease multimorbidity among older adults in selected Indian states: a cross-sectional study. BMJ Open. (2017) 7:e013529. doi: 10.1136/bmjopen-2016-013529, PMID: 28274966 PMC5353268

[73] Oni T McGrath N BeLue R Roderick P Colagiuri S May CR. Chronic diseases and multi-morbidity--a conceptual modification to the WHO ICCC model for countries in health transition. BMC Public Health. (2014) 14:575. doi: 10.1186/1471-2458-14-575, PMID: 24912531 PMC4071801

[74] Arokiasamy P Uttamacharya JK. Multi-morbidity, functional limitations, and self-rated health among older adults in India: cross-sectional analysis of LASI pilot survey, 2010. SAGE Open. (2015) 5:215824401557164. doi: 10.1177/2158244015571640

