# Supplementary material for: Voices of care: unveiling patient journeys in primary care for hypertension and diabetes management in Kerala, India
Source: Front Public Health. 2024 May 22;12:1375227. doi: 10.3389/fpubh.2024.1375227 (PMC11155455; doi:10.3389/fpubh.2024.1375227)
Supplement: Supplementary file 1 [file Data_Sheet_1.docx]

**Supplementary material**

**Table S1: Patient interview topic guide**

| Patient topic guide   1. Can you please talk me through a typical visit with your physician– in the clinic?  - How easy or difficult is it to travel to the clinic? - How friendly are the reception staff at the clinic? - Similarly, what usually happens during visits and in between these visits? [prompt if not mentioned]   [things to ask about] services available, patient-provider interactions, screenings, tests, referrals to specialists, self-management, etc.   - Does your doctor, nurse, community health worker or ASHA follow-up with you after visits? - What do you in case of symptom exacerbation or emergency?  1. In general, how well do you get along with your doctor?   [these prompt questions should be asked if patients don’t mention them in their answer]   - How long have you been seeing this current doctor? How often do you visit   him/her in a year?   - What conditions are you currently seeing your GP for? - How long have you had [this condition]? - How did you get to know about this condition? - Can you describe your journey after you got diagnosed till the point of seeking care? - What are your reasons for seeking care from this facility? - Does the doctor(he/she) know you well as a person? - Is he/she easy to understand most of the time? Do they explain things carefully? - Do you often make decisions about your health together with your doctor? Is that important to you? - How well do they listen to any worries or concerns you might have?  1. Based on your experiences of care at the facility, what are the most important areas for improvement  - What was the best thing about taking treatment? What was the most difficult? - e.g. using simple language, cost issues, access issues to medicines, laboratory and other, interactions with clinic staff]? - Does your doctor/Nurse know about these areas of concern? - [Prompt if No] How comfortable would you feel about talking to your doctor about these areas of concern? - Does your GP or other staff at the clinic ask you for your feedback on your experience of care? [prompt/follow-up question]   If yes: How do they ask you for your feedback?  If no: Is this something you would find useful or important to do on a regular basis?  [PROMPT if ‘quality’ issue not brought up] Thinking back on your answers today, what does ‘good quality of care’ mean to you?   - From your point of view, what are possible reasons/ causes for barriers you have mentioned - What would need to happen to reduce, eliminate, or make it easier to overcome the barriers you mentioned |
| --- |
