# Supplementary material for: Voices of care: unveiling patient journeys in primary care for hypertension and diabetes management in Kerala, India
Source: Front Public Health. 2024 May 22;12:1375227. doi: 10.3389/fpubh.2024.1375227 (PMC11155455; doi:10.3389/fpubh.2024.1375227)
Supplement: Supplementary file 2 [file Data_Sheet_2.docx]

**Table S2:Additional quotes**

| **Themes** | **Illustrative quotes** |
| --- | --- |
| **Approachability and ability to perceive** | *“Three months ago, I began encountering body aches and persistent fatigue. I was neglecting the symptoms, but my sister, who is diabetic, advised me to undergo a blood sugar check. Following her suggestion, I got tested from a private lab, and the results revealed a fasting blood sugar level of 160. However, I'm taking a cautious approach, as once medications are initiated, I will have to take them lifelong. So, I'm avoiding sugar and engaging in regular walks, hoping my blood sugar levels will normalize. As of now, I haven't consulted any medical professionals regarding this matter****.”(45-year old woman with diabetes)***  *As an asthma patient, I suffered from associated problems like weight gain, body swelling, and headache. Consequently, I initially attributed the symptoms I was experiencing to my asthma condition. During that period, my father was severely ill. After his passing, I found myself emotionally and physically weakened, struggling to cope. Eventually, my ability to tolerate these challenges diminished, and I was diagnosed with high blood pressure****.” (51-year-old woman with asthma and hypertension)***  *Twelve years ago, I witnessed my mother-in-law battle diabetes and eventually succumb to it. Back then, I was youthful and full of energy; my focus centered on enjoying good meals and working hard for financial stability. The notion of adhering to a restricted diet never crossed my mind. I believed that diabetes primarily impacted the older adults, and I had never encountered anyone under 50 suffering from it. Even when I began experiencing fatigue and an unquenchable thirst, I dismissed these symptoms as non-serious. Consequently, I was taken aback and shocked when the doctor informed me of my diabetes diagnosis at the age of 38. (****48-year-old woman with diabetes)***  *“I have had tiredness since a long, I have read that diabetes can cause these symptoms; my mother was diabetic. I used to give my blood for testing after taking food, in private labs, whenever I took children for consultation, or when their urine and sputum samples were given. When we go with children, we cannot test fasting sugar. The lab technician tells me it’s not very high, so there is nothing to worry about. I assumed I was alright, until recently when I fell unconscious on the road****.” (53-year-old woman with diabetes and hypertension)*** |
| **Acceptability and ability to seek.** | *“My blood pressure levels are not significantly high, and I don't experience any major issues. With my strict diet and routine, I can effectively manage my condition. I choose to continue visiting the family health centre (FHC). FHC serves for minor ailments such as fever and headache and mild cases of diabetes and hypertension due to the availability of only selected medications and general doctors. I've observed that individuals with multiple health concerns like – high blood sugar, heart issues, high blood pressure, and cholesterol – seldom frequent these small centres”* ***(59-year-old woman with hypertension).***  *“While I receive medications for diabetes from the public health centre, I occasionally undergo comprehensive check-ups and consult with Dr. XXX, a private diabetes specialist. I have consistently sought his expertise and prescribed treatments for various health issues. During my recent visit, Dr. XXX carefully reviewed all my medical records and assured me everything appeared normal. He confidently stated that I was in good health for my age, giving me a comforting pat on the shoulder. This greatly relieved my concerns."(****64-year-old woman with diabetes)***  *“Whenever I encounter discomfort or symptoms such as dizziness, headaches, or shivering due to elevated blood pressure, I consult a specialist doctor in a private hospital and procure medications from a private pharmacy. I revert to using tablets from the public facility once my symptoms subside and I experience relief****.” (44-year-old woman with hypertension).***  *“Government doctors often engage in private practice at their homes during the afternoon. They typically stop issuing tokens at health centres by noon. Their private practice charges patients around 250-400 rupees each, allowing for detailed consultations. They take time to inquire about how we feel when sitting, walking, working, and conduct thorough examinations at home appointments****”* (*62-year-old man with diabetes and hypertension)***  “*When I seek care in private settings, there is a difference in the level of attention and consideration. Perhaps it is because they charge a fee per person. In government facilities, doctors receive the same salary regardless of whether they consult one person or ten. Maybe I feel this is why they don’t do thorough examination or attentive listening.”* ***(55-year-old woman with diabetes)***  *“Experiencing back pain, I arrived at the health centre early in the morning. After securing a token and enduring a lengthy wait, my discomfort was considerable – making it challenging to sit or stand. The doctor inquired about my condition, but he swiftly recommended an X-ray before I could fully explain. Following the X-ray, he swiftly prescribed medication, concluding the consultation within 2 or 3 minutes. He didn't delve into the pain's origin or inquire about its causes, crucial aspects that should have been addressed. No explanations were offered or sought. Disheartened by the experience, I informed my husband that I no longer wish to continue seeking treatment from the same doctor.* *When a doctor lacks the time to listen to my concerns genuinely, I don’t feel like returning to the same doctor”****(45-year-old woman with diabetes)***  *“After being diagnosed with high blood pressure, I briefly tried ayurvedic medicines. I have heard that allopathic medicines are typically lifelong once started, so I attempted Ayurveda. The regimen was too cumbersome, and I discontinued use within a month****". (45-year-old woman with hypertension)***  *" I am not optimistic about a complete cure for diabetes. If I switch to using only insulin and discontinue the tablets, I believe it could benefit my kidneys. I am concerned that the tablets might harm my kidney health."* ***(60-year-old woman with diabetes, and early renal disease).*** |
| **Availability and ability to reach** | *“*  *“The people relying on this institution come from economically disadvantaged backgrounds. Therefore, it is essential to ensure a sufficient supply of medicines. While improvements like infrastructure enhancements such as airport chairs and water facilities have been made, our most critical needs are still unaddressed. The efforts being made are commendable, but it would be highly beneficial if we could receive all necessary medications free of charge. We are willing to patiently wait in line if it means accessing medicines without any cost****.”(FGD)***  *“Certain medications remain unavailable for extended periods; for instance, insulin was absent at the health centre for three consecutive months. On occasion, I acquired these medicines from Jan Aushadhi. Following heart surgery, the doctor prescribed additional medicines worth Rs 2700 per month, distinct from my regular diabetes and hypertension medications. Unfortunately, these medicines are inaccessible at the medical college and the health centre. Moreover, a specific small white tablet for sugar management is consistently unavailable at the health centre, leading me to purchase it from an external pharmacy for the past four months.”* ***(56-year-old woman with diabetes, hypertension, and CVD)***    “*At the FHC, there is a lack of lab facilities. As a result, I visit Neethi Labs, where I can avail of subsidised services. I undergo blood sugar tests and consult a doctor there every two months."(****56-year-old woman with diabetes, hypertension, and CVD)*** |
| **Affordability and ability to pay** | *“We often face irregularities in receiving medicines from the health centre, particularly insulin.... every month. Then, I have no choice but to buy medicines from a private pharmacy when they're unavailable at the FHC. They are costly, and affordability is a big worry. Being a tailor, my income isn't steady, making it hard to manage. When I consider the travel costs and buying medicines monthly, it's a financial challenge*." ***(57-year-old woman with hypertension).***  *“When I cannot obtain medicines from the primary health centre consistently, I end up purchasing the tablets myself. As I am employed, I have limited time and cannot visit every week to check for medicine availability.” (****43-year-old man, with diabetes and hypertension****)* |
| **Appropriateness and ability to engage** | *“Diabetes is a lifelong burden. I think a cure might come when I die (sense of hopelessness). Despite my efforts with vegetables and a healthy diet, sugar levels vary. Even after taking both insulin and tablet, blood sugar remains high. Efforts and various treatments appear futile against this condition, leaving me without a solution.”* *(****67-year-old woman with diabetes and hypertension)***  *“I haven't been given any guidance regarding my diet or physical activity at this facility. However, when I was diagnosed at AA Hospital (a private hospital), the doctor advised me to avoid certain foods like papads, pickles, and salted fish. I've adhered to this advice and have significantly reduced my consumption of these items. Papad is now a rare indulgence for me****.”(59-year-old woman with hypertension)***  *"Seeing long queues of kidney disease patients awaiting dialysis at private hospitals, including both young and old individuals, thinking about the hefty amount of money required for dialysis, its 3000 Rs per day, then transport costs, I always think about my future.,* d*espite my ongoing struggle with diabetes, I haven't shared these concerns with anyone. Already, my body is affected by diabetes!! Whether I take medicines or not, what will happen to me? Will I also have to undergo dialysis?! The sight of others in such circumstances triggers a fear within me.* *I've heard accounts of individuals diagnosed with diabetes at a young age and gradually progressed to needing dialysis within 10-20 years. While some others experienced a rapid onset of elevated sugar levels and kidney complications."* (***60-year-old woman with diabetes and early renal disease)***  *“As far as I comprehend, diabetes is a condition I can effectively manage through dietary restrictions, exercises like walking, and medication. I've successfully refrained from consuming sweets and foods like tubers. Currently, I no longer need to visit the doctor every month. Instead, I monitor my monthly lab results, and if I notice elevated values, I choose to tighten my dietary regimen rather than adjusting my medication dosage.”* ***(57-year-old woman with diabetes)***  *Talking about managing diabetes, controlling our intake of fruits and sweets enables us to manage sugar levels on our own. However, if we encounter other health concerns, such as heart issues, it becomes essential to consult a doctor every month. In my case, I check my sugar levels at an outside(private) laboratory every month. While there may be minor variations in the reported sugar values – sometimes slightly lower and other times marginally higher – I consistently adhere to the prescribed medication (half a tablet) as advised by the doctor. When I feel excessively tired, I may take a whole pill instead of the usual half. Generally, there isn't much else that needs to be done. Doctors typically continue prescribing the same medications. As a result, I do not find it necessary to make monthly visits to the clinic. (****55-year-old man with diabetes, taking medicines for six months)***  *I know how to manage and control my sugar, and I refrain from consuming sweets or chocolate. Over time, I've become attuned to my body's signals and symptoms. Initially, I experienced fear, but now I know when my blood sugar is decreasing, prompting me to adjust my medication and have some juice. Dr K played an important role in teaching me to manage symptoms and guide me on actions to be taken in those circumstances. She ensures that I take medicines regularly and constantly motivates me” (****51-year-old woman with diabetes and CVD)***  *“I manage my diabetes by taking medication twice a day. While a single tablet used to be effective during my private treatment, the tablets provided at the public health centre have a lower dosage, resulting in an 8-hour duration of effectiveness. This necessitates taking two tablets daily – one in the morning and another in the evening. However, due to the increased frequency of dosing, there are occasions when I forget to take one of the tablets.” (****43-year-old man with diabetes and hypertension****).* |
